# Does prolonged labor affect the birth experience and subsequent wish for cesarean section among first-time mothers? A quantitative and qualitative analysis of a survey from Norway

**DOI:** 10.1186/s12884-020-03196-0

**Published:** 2020-10-08

**Authors:** L. C. Gaudernack, T. M. Michelsen, T. Egeland, N. Voldner, M. Lukasse

**Affiliations:** 1 Department of Obstetrics and Gynecology, Rikshospitalet, Oslo University Hospital / Oslo Metropolitan University, College of Applied Sciences, Oslo, Norway; 2 Department of Obstetrics Rikshospitalet, Division of Obstetrics and Gynecology, Oslo University Hospital, Oslo, Norway and Institute of Clinical Medicine, University of Oslo, Oslo, Norway; 3 Biostatistics (BIAS), Norwegian University of Life Sciences, Aas, Norway; 4 Faculty of Health Studies, VID Scientific International Diaconal Specialized University Oslo, Oslo, Norway; 5 Faculty of Health Sciences, Oslo Metropolitan University, Oslo, Norway; 6 Faculty of Health and Social sciences, University of South-Eastern Norway, Notodden, Norway

**Keywords:** First time mothers, Prolonged labor, Birth experience, Wish for a cesarean section in subsequent pregnancies

## Abstract

**Background:**

Prolonged labor might contribute to a negative birth experience and influence first-time mothers’ attitudes towards future pregnancies. Previous studies have not adjusted for possible confounding factors, such as operative delivery, induction and postpartum hemorrhage. We aimed to determine the impact of prolonged labor on birth experience and a wish for cesarean section in subsequent pregnancies.

**Methods:**

A survey including the validated “Childbirth Experience Questionnaire”. First-time mothers giving birth between 2012 and 2014 at a Norwegian university hospital participated. Data from deliveries were collected. Regression analysis and thematic content analysis were performed.

**Results:**

459 (71%) women responded. Women with labor duration > 12 h had significantly lower scores on two out of four sub-items of the questionnaire*: own capacity* (*p* = 0.040) and *perceived safety* (*p* = 0.023).

Other factors contributing to a negative experience were:

Cesarean section vs vaginal birth: *own capacity* (*p* = 0.001) and *perceived safety* (*p* = 0.007). Operative vaginal vs spontaneous birth: *own capacity* (p = 0.001), *perceived safety* (*p* < 0.001) and *participation* (*p* = 0.047).

Induced vs spontaneous start: *own capacity* (*p* = 0.039) and *participation* (*p* = 0.050). Postpartum hemorrhage ≥500 ml vs < 500 ml: *perceived safety* (*p* = 0.002) and *participation* (*p* = 0.031).

In the unadjusted analysis, prolonged labor more than doubled the risk (odds ratio (OR) 2.66, 95%CI 1.42–4.99) of a subsequent wish for cesarean delivery. However, when adjustments were made for mode of delivery and induction, emergency cesarean section (OR 8.86,95%CI 3.85–20.41) and operative vaginal delivery (OR 3.05, 95%CI 1.46–6.38) remained the only factors significantly increasing the probability of wanting a cesarean section in subsequent pregnancies.

The written comments on prolonged labor (*n* = 46) indicated four main themes:

- Difficulties gaining access to the labor ward.
- Being left alone during the unexpectedly long, painful early stage of labor.
- Stressful operative deliveries and worse pain than imagined.
- Lack of support and too little or contradictory information from the staff.

**Conclusions:**

Women with prolonged labors are at risk of a negative birth experience. Prolonged labor per se did not predict a wish for a cesarean section in a subsequent pregnancy. However, women with long labors more often experience operative delivery, which is a risk factor of a later wish for a cesarean section.

## Background

Prolonged labor is associated with an increased risk of complicated deliveries and interventions, such as operative delivery [1, 2], postpartum hemorrhage [3, 4], anal sphincter injury [5], chorioamnionitis and transfer to a neonatal intensive care unit [2]. The WHO defines prolonged labor as active labor that lasts for more than 12 h [6]. In a Norwegian randomized controlled trial, 365 of 3303 (11%) first-time mothers with spontaneous onset of labor had prolonged labor according to the WHO partograph [7].

Studies regarding prolonged labor and birth experience show conflicting results. Some researchers have found that women with long labors had an increased risk of a negative birth experience [8–13], while others did not find such an association [14, 15]. A negative birth experience might lead to women avoiding childbearing [12] or wanting an elective cesarean section in a subsequent pregnancy [16–18]. A negative birth experience can also impair the mental health of women who have recently given birth [19, 20]. As a consequence, the health and development of their children may also be negatively affected [21].

Several cohort studies have investigated the impact of prolonged labor on birth experience using the Childbirth Experience Questionnaire [8–10, 14]. However, in these previous studies, no adjustments were made for confounding factors known to have an impact on birth experience, such as operative deliveries [12, 22, 23], postpartum hemorrhage [24], low Apgar score [25] or induction of labor [26]. In the present study, data were available to adjust for these possible confounders. Many studies have investigated intrapartum events that might predict a subsequent request for cesarean section, such as a negative birth experience or an operative delivery [16, 27, 28]. In a recent systematic review of qualitative research, prolonged labor seemed to be one of several factors contributing to such a request [29]. Nevertheless, we have not found any quantitative studies investigating a possible relation between prolonged labor and a subsequent wish for cesarean section.

Active labor is usually defined as the period from 4 cm dilatation of the cervix until the baby is born [8, 10, 11, 14]. However, there are other aspects of labor progression that may be of importance for the birth experience, such as the duration of early labor (cervix dilated 1–4 cm, often defined as the latent phase). A long latent phase may be associated with an increased risk of both interventions and complications, as well as a negative birth experience [30]. In the present study, the early stage of labor is taken into account, as the start of labor was defined according to the guidelines at the study hospital: a fully effaced cervix with at least 1 cm dilatation in the presence of regular, painful contractions. The study hospital is a part of Oslo University Hospital and these guidelines are still in use at Oslo University Hospital.

The aim of this study was to determine the association between: 1) prolonged labor and a negative birth experience and 2) prolonged labor and a wish for cesarean section in later pregnancies. We hypothesized that prolonged labor is associated with a negative birth experience and a wish for cesarean section in subsequent pregnancies.

## Methods

We have used data from the database *First-time mothers and birth experience,* including first time mothers at term with one fetus in cephalic position, at a university hospital in Norway that had 2700 births per year. The respondents had to be able to read and write in Norwegian to be included. Women who gave birth by elective cesarean section were excluded. The data suitable for our aim was collected in two periods, from October 2012 to May 2013 and from April 2014 to October 2014.

Eligible women were sent a questionnaire by mail 1–2 months after birth. After one reminder, 459 of 646 (71%) women responded. Due to mainly administrative reasons, no questionnaires were sent out between May 2013 and April 2014.

**Fig. 1:**
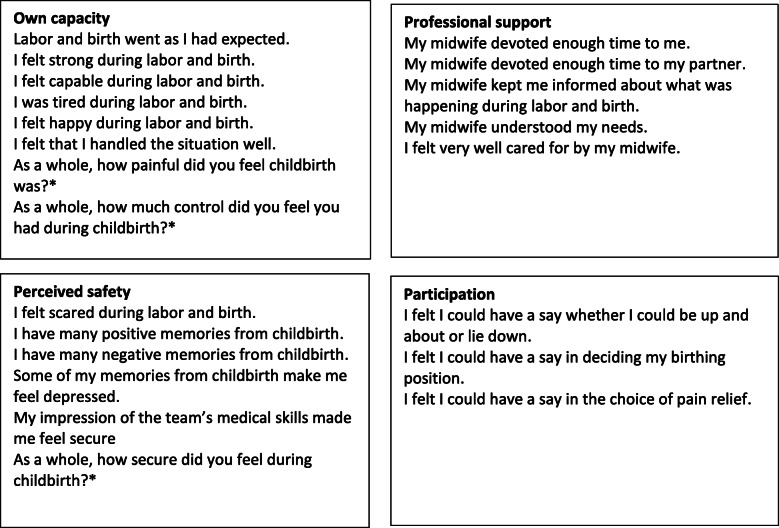
The four categories of the questionnaire with respective questions. In the present study, the following question was added: *To what extent do you agree with the statement “If I decide to have more children, I want to be delivered by cesarean section”?*. Each question has four answering options (totally agree, mostly agree, mostly disagree, disagree). *Measured using visual analogue scale

The present study investigated the effect of labor duration on birth experience, measured by the Childbirth Experience Questionnaire (CEQ) [8]. The questionnaire is validated in Swedish and translated into Norwegian. We used the Norwegian version. CEQ consists of four categories: *perceived safety, professional support, own capacity* and *participation.* The response format was a 4-point Likert scale with the following response options: 1 (totally agree), 2 (mostly agree), 3 (mostly disagree) and 4 (totally disagree). Memory of labor pain, sense of security and control were assessed with a visual analogue scale (VAS) ranging from 1 to 100 [8]*.* In addition to the 22 questions on the questionnaire, we added the following question: To what extent do you agree with the statement “If I decide to have more children, I want to be delivered by cesarean section”? The same Likert scale that was used in the rest of the questionnaire was used for this question (Fig. 1).

### Statistical methods

Statistical analyses were performed using SPSS statistics version 24.0 (IBM; Armonk, NY, USA). In Table 1, the study population data were placed alongside data from the Norwegian Birth Registry. When available, data from first-time mothers with spontaneous and induced labor were used; otherwise, we used data from all women giving birth in Norway in 2014.

**Table 1:** Maternal, fetal and labor characteristics

	Study population Primiparous at term, induced or spont. start of labor (*n* = 459)	Medical Birth Registry, Norway, 2014 a All deliveries *n* = 59,065 b All planned vaginal deliveries (*n* = 54,946) c Primiparous spont. start (*n* = 14,239) d Primiparous spont. start, vag delivery (*n* = 12,954)
**Maternal characteristics**		
Maternal age mean, yrs	32	29 a
Cohabiting or married Missing	439 (96%) 9 (2%)	55,041 (93%) a
BMI median Missing	22.0 43 (9.4%)	24.2 a
**Fetal characteristics**		
Gestational age, days	283 (SD = 9)	275 (SD = 14) a
Mean birth weight, g	3438	3489 a
5 min Apgar score < 7 Missing	3 (0.7%) 5 (1.1%)	1059 (1.8%) a
**Onset of labor**		
Spontaneous	346 (75%)	42,848 (78%) b
Induced	113 (25%)	12,098 (22%) b
**Mode of delivery**		
Normal vaginal	296 (64%)	10,093 (71%) c
Operative vaginal	114 (25%)	2861 (20%) c
Cesarean section	49 (11%)	1285 (9%) c
**Labor characteristics**		
Anal sphincter tears	7 (1.5%)	406 (3.1%) d
Missing	5 (1.1%)	
Episiotomy	155 (34%)	4469 (34%) d
Bleeding ≥500 ml	109 (24%)	15,802 (27%) a

In our study, labor duration was measured from the start of labor was documented by a midwife in the patient’s medical chart, until birth. The labor duration was not normally distributed. Moreover, transformation to normality was not possible. This is typical for duration data of this kind. We therefore used labor lasting more than 12 h to indicate prolonged labor. This representation of labor duration simplifies interpretation. The mean score for the four subcategories of the CEQ was reported for each group (i.e., duration more or less than 12 h) as has been done in comparable studies [8–11]. In Table 2, we compared the prevalence of complications and interventions depending on labor duration, using the chi-square test. The proportion of women who wished to be delivered with a planned cesarean section in a future pregnancy was also compared between the two groups. Mode of delivery among women with such a wish was indicated.

**Table 2:** Characteristics of labors lasting more than 12 h compared to labors lasting 12 h or less

	Labor duration > 12 h *n* = 85 *n* (%)	Labor duration ≤ 12 h *n* = 373 *n* (%)	*p*-value
< 5 cm cervical dilatation at labor start	83 (97.6)	263 (70.5)	< 0.001
Induction	21 (24.7)	92 (24.7)	ns
Oxytocin augmentation	84 (98.8)	205 (55)	< 0.001
Epidural analgesia	82 (96.5)	218 (58.4)	< 0.001
Spontaneous vaginal birth	35 (41.2)	260 (69.7)	< 0.001
Operative vaginal birth	32 (37.6)	82 (22)	0.003
Intrapartum cesarean section	18 (21.2)	31 (8.3)	0.001
Bleeding ≥500 ml	33 (38.8)	76 (20.4)	0.001
Sphincter rupture	1 (1.6)	6 (1.2)	ns
5 min Apgar score < 7	2 (2.4)	1 (0.3)	ns
Wants a caesarean next time	18 (21.2) a	34 (9.1) b	0.003

a: 4 CS, 11 opr vag, 3 spontaneous b: 15 CS, 7 opr vag, 12 spontaneous

In Table 3, linear regression analysis was used, with the mean scores for each category of the questionnaire as outcome variables. However, for the category *professional support,* linear regression could not be performed, as the residuals were not normally distributed and transformation to normality not possible. Median scores were used for this category since the distribution was asymmetric. Table 4 presents questions for which women with prolonged labor scored significantly lower. *P*-values were calculated using the chi-square test.

**Table 3:** Intrapartum factors with impact on CEQ scores in subcategories

	Own capacity	Professional support	Perceived safety	Participation
All participants	Mean (SD) 2.66 (0.53)	Median (Quart) 4.00 (3.60,4.00)	Mean (SD) 3.30 (0.59)	Mean (SD) 3.36 (0.68)
**Labor start**				
Induced labor start (*n* = 113)	2.51 (0.54)	4.00 (3.60,4.00)	3.22 (0.60)	3.23 (0.66)
Spontaneous start of labor (*n* = 346)	2.70 (0.52)	4.00 (3.60,4.00)	3.33 (0.58)	3.40 (0.68)
*p*-value	0.001	ns	0.108	0.024
a Adjusted *p*-value	0.039		0.696	0.050
**Labor duration**				
Labor duration > 12 h (*n* = 85)	2.51 (0.55)	4.0 (3.60,4.00)	3.12 (0.65)	3.46 (0.56)
Labor duration < 12 h (*n* = 373)	2.69 (0.53)	4.0 (3.60,4.00)	3.34 (0.56)	3.33 (0.70)
*p*-value	0.004	ns	0.001	0.125
a Adjusted *p*-value	0.040		0.023	0.068
**Mode of delivery**				
Cesarean section (*n* = 49)	2.30 (0.48)	3.80 (3.40,4.00)	2.96 (0.66)	3.30 (0.59)
Vaginal delivery (*n* = 410)	2.70 (0.52)	4.0 (3.60,4.00)	3.34 (0.56)	3.37 (0.69)
*p*-value	< 0.001	ns	< 0.001	0.541
a Adjusted *p*-value	0.001		0.007	0.707
Operative vaginal delivery (*n* = 114)	2.54 (0.48)	4.0 (3.45,4.00)	3.14 (0.61)	3.25 (0.74)
Spont. vaginal delivery(*n* = 296)	2.76 (0.53)	4.0 (3.60,4.00)	3.42 (0.53)	3.41 (0.66)
*p*-value	< 0.001	ns	< 0.001	0.042
b Adjusted *p*-value	0.001		< 0.001	0.047
**Postpartum hemorrhage**				
≥ 500 ml (*n* = 109)	2.52 (0.51)	3.80 (3.40,4.00)	3.09 (0.63)	3.24 (0.71)
< 500 ml (*n* = 350)	2.70 (0.53)	4.00 (3.60,4.00)	3.37 (0.56)	3.40 (0.66)
*p*-value	0.002	ns	< 0.001	0.031
*Adjusted *p* -value	0.118		0.007	0.031

a Linear regression adjusted for all other values in the table. Vaginal delivery includes both operative and spontaneous

b Cesarean section excluded, linear regression adjusted for induction, labor > 12 h, Bleeding ≥500 ml

**Table 4:** Scores for the sub questions from the categories perceived safety and own capacity that differ significantly depending on labor duration

	Labor duration > 12 h *n* = 85				Labor duration ≤12 h *n* = 373				*p*-value
	Totally agree	Partly agree	Partly disagree	Totally disagree	Totally agree	Partly agree	Partly disagree	Totally disagree	
Labor and birth went as I had expected	1 (1.2%)	38 (45.2%)	21 (25%)	24 (28.6%)	35 (9.4%)	217 (58.2%)	79 (21.2%)	42 (11.3%)	< 0.001
I was tired during labor and birth	54 (63.5%)	25 (29.4%)	6 (7.1%)	0	162 (43.4%)	138 (37%)	56 (15%)	17 (4.6%)	0.003
I felt capable during labor and birth	36 (42.4%)	37 (43.5%)	7 (8.2%)	5 (5.9%)	169 (45.3%)	166 (44.5%)	34 (9.1%)	4 (1.1%)	0.039
Some of my memories from childbirth make me feel depressed	8 (9.4%)	14 (16.5%)	20 (23.5%)	43 (50.6%)	15 (4%)	37 (10%)	90 (24.3%)	229 (61.7%)	0.046
I have many negative memories from childbirth	7 (8.2%)	30 (35.3%)	36 (42.4%)	12 (14.1%)	16 (4.3%)	91 (24.5%)	152 (41%)	112 (30.2%)	0.008
I felt that I handled the situation well	41 (48.8%)	40 (47.6%)	1 (1.2%)	2 (2.4%)	235 (63%)	117 (31.4%)	18 (4.8%)	3 (0.8%)	0.010
I have many positive memories from childbirth	26 (30.6%)	31 (36.5%)	23 (27.1%)	5 (5.9%)	148 (39.9%)	156 (42%)	58 (15.6%)	9 (2.4%)	0.020

*P*-values are calculated using the chi square test

In Table 5, logistic regression analysis was used to determine if there was a significant association between prolonged labor and the wish for a cesarean section in a subsequent pregnancy. The four response categories for the statement “If I decide to have more children, I want to be delivered by cesarean section” were recoded into two categories: *agree* consists of *agree* and *mostly agree* responses, and *disagree* consists of *disagree* and *mostly disagree*. To show the relation between CEQ scores and agreement with wanting a cesarean section in a subsequent pregnancy, we performed Mann-Whitney U tests.

**Table 5:** Intrapartum factors with an impact on a subsequent wish for cesarean section

	Wants a CS next time* n* (%)	Does not want a CS next time *n* (%)	OR (95%CI) Unadjusted	*p*-value Unadjusted	OR(95%CI) Adjusted	*p*-value Adjusted
**Labor duration** (3 missing)						
Labor ≤ 12 h (ref) *n* = 371	34 (9.2)	337 (90.8)				
Labor > 12 h *n* = 85	18 (21.2)	67 (78.8)	2.66 (1.42, 4.99)	0.002	1.80 (0.90, 3.58)	0.096
**Labor start** (2 missing)						
Spontaneous start (ref) *n* = 346	30 (8.7)	315 (91.3)				
Induced start *n *= 113	22 (19.6)	90 (80.4)	2.57 (1.41, 4.67)	0.002	1.63 (0.83, 3.19)	0.155
**Mode of delivery** (2 missing)						
Spontaneous vaginal birth (ref) *n* = 295	15 (5.1)	280 (94.9)				
Operative vaginal birth *n* = 114	18 (15.8)	96 (84.2)	3.50 (1.70, 7.22)	0.001	3.05 (1.46, 6.38)	0.003
Emergency cesarean section *n* = 48	19 (39.6)	29 (60.4)	12.23 (5.62, 26.61)	< 0.001	8.86 (3.85, 20.41)	< 0.001

Explanation of table headings: *Wants a CS next time*; women totally or partly agreeing to the statement: “*If I decide to have more children, I want to be delivered by cesarean section”*. *Does not want a CS next time*: women totally or partly disagreeing to this statement. The two rightmost columns are based on multiple logistic regression adjusted for the other variables in the table

Based on previous publications, we identified labor induction [26], operative delivery [23] and postpartum bleeding [24] as possible confounders for a negative birth experience. Operative delivery and labor induction [16, 27, 28] were identified as possible confounders for a wish to be delivered by cesarean section in subsequent pregnancies.

**Fig. 2:**
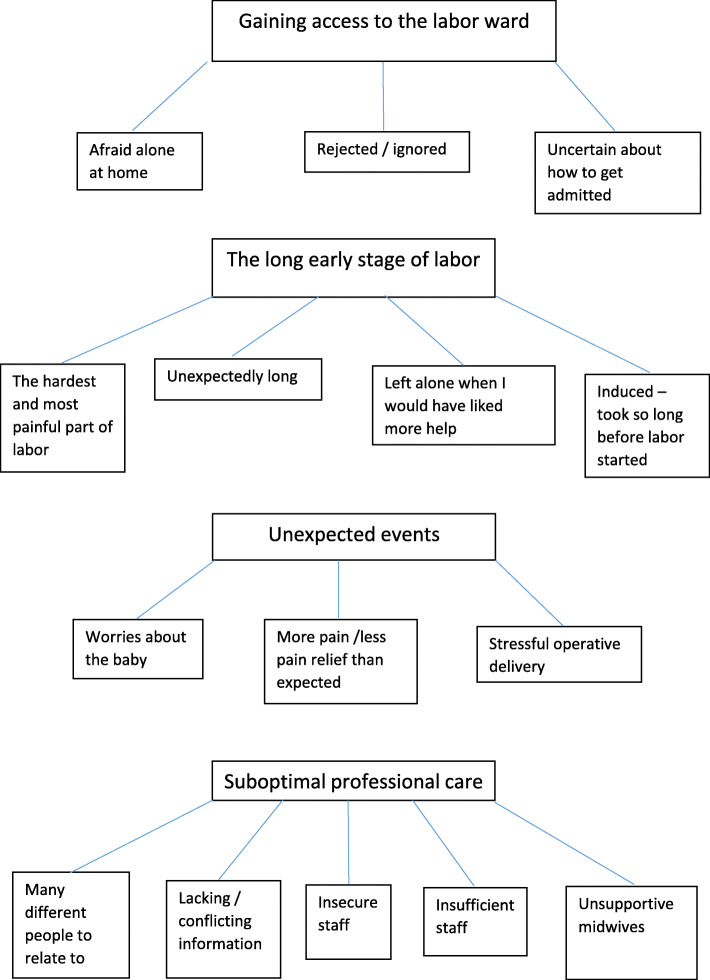
Themes and sub themes from the written comments on the questionnaire

**Fig. 3:**
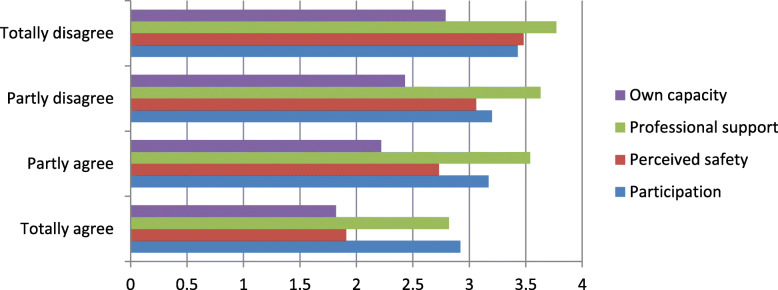
Women totally or mostly agreeing with the statement: *“If I decide to have more children, I want to be delivered by cesarean section”* scored significantly lower in all four subcategories of the CEQ than women totally or mostly disagreeing with this statement (*p*-value < 0.003 for all categories)

On the last page of the questionnaire, the respondents had the opportunity to write their own comments; 283 women did so, and 46 described a long labor as a negative experience. We have analyzed these 46 comments using thematic analysis as described by Braun and Clarke [31] (Fig. 2). The thematic analysis was performed to identify meaningful patterns and to capture the themes embedded in the participants’ experiences. We used the six steps described by Braun and Clarke; the process was started with the authors (LCG and ML) reading the comments several times to familiarize themselves with the content. Then, initial codes were created, meaning that the authors found statements that were assumed to have the same or similar meaning [31]. In the third step, potential themes were developed by bringing statements with similar content together. In step four, the themes were revised. All statements within each theme and subtheme were revised by LCG and ML until consensus was reached on which themes to present. The result is a thematic map shown in Fig. 2. In step five, the themes and subthemes were given names considered by the authors to capture the content of the written comments. Step six presents the results [31] (Fig. 3).

### Ethical considerations

The Regional Committee of Medical and Health Research Ethics concluded that the study was a quality improvement project and that ethical approval was not necessary (REC number: 2011/1581 D/14.10.2011). The Personal Data Officer (PVO) at the study hospital gave permission for this study to be performed.

## Results

The study population was older and had more inductions of labor, cesarean sections and vaginal operative deliveries compared to the population from The Medical Birth Registry of Norway (Table 1). There were significantly more interventions and complications among women with prolonged labor (defined as the active phase of labor lasting for more than 12 h). Fifty of 85 women with prolonged labor were delivered operatively. Fifteen of these 50 women agreed they would wish for a planned cesarean section if they would have more children (Table 2).

Compared to women with a labor duration of less than 12 h, those with a labor duration of more than 12 h scored significantly lower in the categories of *own capacity* and *perceived safety,* both in the unadjusted model and after adjustment for labor induction, mode of delivery and postpartum hemorrhage (Table 3). For the categories *professional support* and *participation,* there were no significant differences. Table 4 shows the mean scores for questions in the categories of *own capacity* and *perceived safety*. The answers to three of these questions suggest that women with prolonged labor had more negative memories from their birth, and they were also more likely to disagree with the statement *“Labor and birth went as I expected”*. The answers to two of the other questions indicate that women with prolonged labor were less confident in their own capability. They were also more likely to agree that they were tired during labor and birth.

After adjustment for confounding variables, induction of labor seemed to have a negative effect on *own capacity* and *participation*. Compared to vaginal birth, intrapartum cesarean section was associated with lower scores in the categories *own capacity* and *participation.* Compared to spontaneous birth, operative vaginal birth was associated with lower scores for all categories except for *professional support.* Postpartum hemorrhage greater than 500 ml was associated with lower scores for the categories *perceived safety* and *participation.*

In the unadjusted analysis, women with prolonged labor were more than twice (OR 2.66 95%CI 1.42–4.99) as likely to agree with the statement “*If I decide to have more children, I want to be delivered by cesarean section”* However, this effect disappeared when adjusting for emergency cesarean section, operative vaginal delivery and induction (Table 5). After these adjustments, the only factor significantly increasing the odds of wishing to be delivered by cesarean section in a subsequent pregnancy was operative delivery. The risk of a future wish for cesarean section was almost nine times higher for women delivered by intrapartum cesarean section and three times higher for those with an operative vaginal delivery (Table 5). Women totally or mostly agreeing with the statement: *“If I decide to have more children, I want to be delivered by cesarean section”* scored significantly lower in all four subcategories of the CEQ than women totally or mostly disagreeing with this statement (*p*-value < 0.003 for all categories).

In the qualitative analysis of the written comments, we identified four main themes:

**Gaining access to the labor ward** was the first theme. Some women stated that it was frightening being home alone during labor. When contacting the hospital, they felt rejected and ignored and were uncertain of how to be admitted. This theme is accurately described by the following quote: “*I was at home for 48 hours with intense contractions before labor started. I wish the hospital could have helped me with pain relief and/or short-term appointments so that I wouldn’t feel that nothing was happening and that there was no progress despite intense pain and no sleep”.*

The second theme was **the long early stage of labor***,* which by some women was described as unexpectedly long and the hardest and most painful part of the birth. Women felt left alone in a situation when they needed more help. This theme is illustrated by the following quote: *“When my labor was induced, the process took a very long time, and I had a lot of different midwives to relate to before the active stage of labor started. I also felt left alone for long periods of time after induction started and that I got contradictory information. I experienced the most intense pain before I went into active labor because I had so little control and I had no breaks between the contractions.”*

The third theme was **unexpected events.** The participants described stressful operative deliveries, worries about the baby’s health and more pain and less pain relief than expected. We think this statement is representative of this theme: *“The most negative thing during childbirth was towards the end, when I pushed strongly for a long time and the baby did not come out. It seemed hopeless at times. Finally, our boy was born by vacuum. I was also afraid that the long expulsion phase would hurt the boy”.*

The fourth theme was **suboptimal professional care:** there were many different people to relate to, information that was conflicting or lacking, and the staff were sometimes insecure or insufficient. The difference between supportive and unsupportive midwives is illustrated by the following statement: *“The midwives I met were very different. The midwife who welcomed me made me very scared and insecure, was rude, did not greet me and did not understand that I was afraid that I would have to give birth alone when my husband suddenly became ill. Midwife 2, who was with me most of the time, was a dream; she was considerate, made me feel safe and made me believe that I could do this, while midwife 3 did not know my name, did not read my medical chart, talked over my head and never informed me about what was happening.”*

## Discussion

Women with prolonged labor scored significantly lower on the birth experience sub-items: *own capacity* and *perceived safety*. In addition, operative delivery, induction of labor and postpartum hemorrhage contributed to a negative birth experience. In the unadjusted analysis, prolonged labor more than doubled the odds ratio for a request for a cesarean delivery in subsequent pregnancies. When adding adjustment for operative delivery and induction; intrapartum cesarean section and operative vaginal delivery remained the only independent predictors of a request for cesarean delivery in subsequent pregnancies. Intrapartum cesarean section and operative vaginal delivery increased the probability by nine and three times, respectively. To our knowledge, the present study is the first to show the association between a low CEQ score and a subsequent wish for cesarean section. Women agreeing or mostly agreeing that they would wish to be delivered by a cesarean section in a subsequent pregnancy scored significantly lower in all four subcategories of the questionnaire.

A strength of the present study is that we used a validated questionnaire (CEQ) [8]. A possible limitation is that that the CEQ is not validated in Norwegian. However, it has been validated in Swedish and we consider the obstetric care in Norway and Sweden to be comparable and the populations in our two countries to be quite similar. The finding that women with prolonged labor had a more negative perception of labor is consistent with results from unadjusted analyses in previous studies using the CEQ and defining prolonged labor as a duration of > 12 h [8–11].

In contrast to these studies, we were able to adjust for possible confounders, which we consider a strength. Another strength of our study is that we analyzed written comments and received information beyond the CEQ scores. However, a possible weakness is that women were not asked directly about prolonged labor, and only 46 of the respondents commented on this topic. Thus, the qualitative data regarding prolonged labor must be interpreted with caution. In our opinion, it is an advantage that the definition of active labor was wider and more in line with women’s own definition than in previous studies. This enabled us to identify a larger number of women with prolonged labor. The women experienced that they were in active labor from this time point and the personnel at the labor ward took care of the women as if they were in active labor. The guidelines therefore affected how the women were managed, which again probably affected the women’s birth experience. Ideally, we would prefer to include duration of labor based on the women’s own perception. Unfortunately, such data were not available. Another weakness of our study is that prolonged labor might be associated with lower Apgar scores and a higher prevalence of anal sphincter tears. We did not have sufficient sample size to test for a difference between groups.

A possible weakness of the CEQ is that questions about the latent phase/early stage of labor are lacking. In addition, the question “*labor went as I expected”* is difficult to interpret, as the answer depends on whether the woman had positive or negative expectations. To evaluate the effect of labor duration on birth experience, questions about whether labor progressed as expected would have been useful.

Women undergoing induction of labor are more likely to experience the first stage of labor as prolonged. In a survey investigating the birth experience of induced women, the main contributor to a negative experience was a longer time delay between the start of the induction and delivery. Furthermore, 35% of women in the induction group were not satisfied with the information they received about the induction prior to the procedure [26].

This is consistent with our results indicating that induced women had a more negative birth experience. Even after adjustments were made for operative delivery, prolonged labor and postpartum hemorrhage> 500 ml, induced women reported significantly lower scores for the categories of *own capacity* and *participation,*

Many of the written comments on the questionnaire address the prolonged latent phase or first stage of labor, difficulties with coping with pain at home and not being allowed access to the hospital. These themes were not covered by our questionnaire. The manner in which professional care was perceived could be difficult to respond to in the CEQ because most of the questions regard the midwife as *one* person, but women with long labor processes must relate to many different midwives and doctors. This is a possible weakness in the questionnaire, which was mentioned by some of the respondents, and might explain why many women chose to provide written comments on professional care.

One of the main themes from the written comments in our study was “unexpected events”; some women had stressful operative deliveries, were afraid that the baby would be hurt and experienced more pain than expected. In addition, according to some of the responses in our survey contributing strongly to a negative experience were: not feeling capable and having many negative memories from childbirth. These findings are consistent with the findings of a survey on birth experience by Nystedt et al. [12].

Our respondents commented on how labor was unexpectedly long and how they experienced more pain and less pain relief than expected, which confirms the findings in a qualitative study by Nystedt et al. [13]. Nystedt concluded: “women with prolonged labor are more dependent on their caregivers than are women without prolonged labor. They have a special need for extra support and encouragement during delivery as well as increased nursing and midwifery care” [13]. Our respondents too wanted more help from the midwives, especially in the early stage of labor. Some midwives were described as unsupportive and provided insufficient and conflicting information.

Growing evidence shows that a negative birth experience in the first pregnancy is associated with a subsequent wish for a cesarean section [16, 27, 28, 32]. According to Nystedt et al. [12], women with prolonged labor were more likely to agree that difficulties during birth affected them for life, and the experience made them decide not to have any more children. In our study, when adjusting for cesarean section, operative vaginal delivery and induction of labor, prolonged labor no longer was a risk factor of a later wish for cesarean section. Emergency cesarean section and operative vaginal delivery remained the only significant risk factors for a later wish for a cesarean section. This analysis [12] is in line with our finding that out of 52 women agreeing or mostly agreeing they would want a cesarean next time, 19 were delivered by cesarean and 18 by operative vaginal delivery in their first pregnancy (Table 2).

A Swedish prospective cohort study found that long latent phases were strongly associated with a negative birth experience [25]. The qualitative data from our study indicate that for some women, suboptimal care during this early phase of labor made the birth experience negative. In a Scottish survey among 730 first-time mothers, being turned away from the hospital was a predictor of a negative birth experience [33]. According to NICE guidelines, primiparas and their partners can benefit from pre-labor classes teaching them that the first labor lasts longer, especially the latent phase, and how to cope with those early hours of labor at home [34]. All first-time parents should be offered such classes [34]. Studies show that women who have received extra support to stay at home in the first stage cope better and are admitted during more advanced labor. However, when admitted to the hospital, these women have the same rate of interventions as women admitted earlier in labor [35, 36]. This is in line with the notion that a long latent phase itself, rather than whether the woman stays at home, might explain the increase in interventions and complications [35]. A prospective cohort study showed that women are highly confident that their midwife strongly contributes to a positive birth experience [37]. The support and attention midwives give women in labor, even in the latent phase, is of great importance [13, 35, 37].

## Conclusions

Women with prolonged labor (duration > 12 h) are at risk of a negative birth experience. These women expressed lack of attention and support. Prolonged labor per se did not predict a wish for a cesarean section in a subsequent pregnancy. However, women with long labor more often experienced operative deliveries, which was a risk factor of a later wish for a cesarean section. Low CEQ scores seem to predict a higher probability of a wish for cesarean delivery in next pregnancy.

## Data Availability

Data are regarded as sensitive by the Study Hospital’s Personal Data Officer (PVO) and are therefore stored in the hospital database in a password protected area. The data that support the findings of this study are available, but restrictions apply to the availability of these data, which were used under license for the current study, and so are not publicly available. Data are however available from the authors upon reasonable request and with permission of PVO.

## Abbreviations

WHO: World Health Organization
PVO: Personal Data Officer
REC: Regional Committee for Health and Research Ethics
CS: Cesarean section
CEQ: Childbirth Experience Questionnaire
